# Impact of concomitant use of pazopanib and gastric acid suppressants on progression-free survival and safety in patients with sarcoma: a retrospective study

**DOI:** 10.1186/s40780-025-00477-8

**Published:** 2025-08-18

**Authors:** Tatsuya Isezaki, Hitomi Yuyama, Osamu Yasumuro, Yasutomo Miyaji, Ryohkan Funakoshi

**Affiliations:** 1 Department of Pharmacy, Kameda Medical Center, 929 Higashi-Cho, Kamogawa, Chiba 296-8602 Japan; 2 Department of Oncology, Kameda Medical Center, 929 Higashi-cho, Kamogawa, Chiba 296-8602 Japan

**Keywords:** Histamine H2 receptor antagonist, Pazopanib, Proton pump inhibitor, Sarcoma

## Abstract

**Background:**

Pazopanib (PAZ) is an oral multi-kinase inhibitor used in the treatment of advanced soft tissue sarcoma. Gastric acid suppressants such as proton pump inhibitors (PPIs) and H2 receptor antagonists (H2RAs) may reduce PAZ absorption by increasing gastric pH, potentially affecting its efficacy. This study aimed to evaluate the impact of concomitant use of acid suppressants on progression-free survival (PFS) and safety in patients with soft tissue sarcoma.

**Methods:**

This retrospective study included patients with advanced or metastatic soft tissue sarcoma who were treated with PAZ at a single institution between 2015 and 2022. Patients were divided into two groups: those who received PAZ with concomitant acid suppressants (AS combination group) and those who did not (non-AS group). The primary outcome was PFS. Kaplan–Meier curves were used to estimate survival, and group differences were compared using the log-rank test. Multivariable Cox proportional hazards regression was performed to adjust for confounding factors.

**Results:**

A total of 99 patients were included (77 in the AS combination group, 22 in the non-AS group). The median PFS was 116 days in the AS combination group and 403 days in the non-AS group (hazard ratio [HR]: 1.42; 95% confidence interval [CI]: 0.68–2.85; *P* = 0.361). No statistically significant difference in PFS was observed. Adverse events of any grade occurred in 84% of patients in the AS combination group and 68% in the non-AS group. Grade ≥ 3 adverse events occurred in 33 patients (43%) in the AS combination group and 9 patients (41%) in the non-AS group.

**Conclusions:**

In our cohort of sarcoma patients, the concomitant use of acid-suppressive agents was not associated with a statistically significant difference in PFS. However, the substantial numerical difference in median PFS observed between the groups (403 days vs. 116 days), coupled with the study's limited sample size, suggests a potentially clinically meaningful negative effect that warrants caution and further investigation in larger, prospective studies. Our findings, therefore, do not rule out a detrimental interaction and underscore the need for careful consideration when co-prescribing these agents with pazopanib in this patient population.

## Background

Malignant soft tissue tumors originate from or differentiate into soft tissues [1]. According to a National Cancer Registry-based analysis in Japan, the age-adjusted incidence rate of soft tissue sarcoma was 3.22 per 100,000 in males and 2.70 per 100,000 in females between 2016 and 2019, accounting for less than 1% of all cancers [2]. Treatment typically includes surgery, chemotherapy, and radiotherapy. Traditionally, chemotherapy has comprised doxorubicin hydrochloride and ifosfamide. In 2012, pazopanib hydrochloride (PAZ), a multi-kinase inhibitor targeting vascular endothelial growth factor receptors, Platelet-Derived Growth Factor Receptors, and c-Kit, was approved in Japan for the treatment of malignant soft tissue tumors [3].

Co-administration of PAZ with proton pump inhibitors (PPIs) has been shown to reduce drug exposure by decreasing AUC and C_max [4]. This interaction is based on pazopanib's pH-dependent solubility; it becomes practically insoluble as gastric pH rises above 4.0 [5]. The Japanese label recommends avoiding PPIs when possible. Nevertheless, acid suppressants such as PPIs and histamine H2 receptor antagonists (H2RAs) are frequently prescribed for cancer patients for symptom control or prophylaxis against gastrointestinal bleeding. H2RAs, unlike short-acting antacids, produce a sustained elevation of gastric pH for 10–12 h, creating a theoretical basis for a clinically relevant interaction [6]. This theoretical concern is supported by recent real-world evidence from a large pharmacovigilance study, which found that H2RA co-administration was associated with reduced pazopanib exposure, similar to PPIs [7].

The clinical significance of this potential interaction with H2RAs is, however, debated, with evidence suggesting the impact may differ by tumor type. While some studies reporting no significant clinical impact have primarily focused on renal cell carcinoma patients [8], this contrasts sharply with findings from a large analysis of clinical trial data specifically in soft tissue sarcoma. This analysis found that concomitant use of acid-suppressive agents, including H2RAs, was associated with significantly shorter progression-free survival (PFS) [9]. It is also noteworthy that a Japanese study including both sarcoma and renal cell carcinoma patients found no significant effect of H2RA use on pazopanib trough concentrations [10], though the precise clinical significance of this pharmacokinetic observation remains to be fully elucidated.

This pattern in the literature suggests the clinical consequences of the pazopanib-AS interaction may be tumor-type specific. A potential mechanism for this is the difference in therapeutic targets; the suggested minimum trough concentration for pazopanib efficacy is higher in soft tissue sarcoma (≥ 27 mg/L) than in renal cell carcinoma (≥ 20.5 mg/L) [11]. Therefore, a modest reduction in drug exposure, even if not affecting trough levels to a statistically significant degree in all studies, might negatively impact outcomes specifically in sarcoma patients. Furthermore, in a post-marketing surveillance study of PAZ in Japan [12], the impact of acid suppressants (AS) was not specifically examined.

Given the conflicting evidence and the data suggesting a particular vulnerability for sarcoma patients, this study aimed to evaluate the impact of acid suppressants on PAZ treatment in patients with soft tissue sarcoma. Specifically, the primary objective was to assess the effect of acid suppressant use on PFS. As a secondary objective, we examined the incidence of adverse events (AEs), including gastrointestinal toxicities, that occurred during the observation period. In addition, we conducted subgroup analyses comparing PPI and H2RA to clarify any differential effects.

## Methods

This retrospective analysis was conducted using the electronic medical record system of our institution. Patients diagnosed with sarcoma who initiated PAZ treatment at our hospital between November 1, 2012 and January 31, 2020, were included in this study. Medical records were reviewed up to March 30, 2023, as the final date of data collection.

Patient background factors such as height, weight, age, sex, body mass index (BMI), diagnosis, disease stage, recurrence status, treatment history, PAZ dosage and timing of administration, concomitant use of PPIs or H2RA, use of cytochrome P450 (CYP) 3A4 inhibitors (ketoconazole, itraconazole, clarithromycin, ritonavir, voriconazole) or inducers (rifampin, carbamazepine, phenytoin), presence of gastrointestinal disorders, Eastern Cooperative Oncology Group performance status (PS), and histological classification were collected from the electronic medical records.

Information on the concomitant use of PPIs or H2RAs was obtained from prescriptions recorded in the hospital’s electronic medical records, which include all inpatient and outpatient medications prescribed within the institution. Medications brought in by patients at the time of hospital admission (i.e., self-medicated or prescribed at another institution but confirmed during medication reconciliation) were also reviewed and included when available. However, over-the-counter (OTC) medications that were not recorded in the medical records were not captured in this analysis.

Although height, weight, and BMI are interrelated variables, they were analyzed individually to identify any potential differences in baseline characteristics between groups.

The primary endpoint was PFS of patients whose treatment comprised PAZ and AS (AS combination group) and that of patients whose treatment comprised PAZ alone (non-AS group).

The secondary endpoint was treatment safety, which was evaluated by analyzing AEs related to PAZ. For this purpose, AEs were classified into two categories based on clinical relevance and the PAZ drug label: serious AEs potentially associated with PAZ, and gastrointestinal toxicities. The following events were designated as serious AEs associated with PAZ, based on known safety concerns described in the Japanese package insert and prior literature: Liver failure, Aspartate Aminotransferase (AST) increased, Alanine Aminotransferase (ALT) increased, blood bilirubin increased, Gamma-Glutamyl Transferase (GGT) increased, hypertension and hypertensive crisis, left ventricular systolic dysfunction, QT corrected interval prolonged, ventricular arrhythmia, cardiac troponin I increased, angina pectoris, ischemic stroke, transient ischemic attacks, myocardial ischemia, venous thrombosis, pulmonary embolism, cerebral hemorrhage, hemoptysis, gastrointestinal bleeding, hematuria, bronchopulmonary hemorrhage, epistaxis, gastrointestinal perforation, gastrointestinal fistula, thyroid dysfunction, nephrotic syndrome, proteinuria, infectious diseases, delayed wound healing, interstitial pneumonia, thrombophilia, reversible posterior leukoencephalopathy syndrome, pancreatitis, retinal detachment, hair color changes, palmar-plantar erythrodysesthesia syndrome, gastric ulcer, duodenal ulcer, reflux esophagitis, acute and chronic gastritis. In addition, gastrointestinal toxicities were examined separately as clinically relevant endpoints because of their frequency and potential impact on treatment continuity. The following symptoms were included: Reflux esophagitis, acute and chronic gastritis, nausea, vomiting, diarrhea, and stomach pain. Each adverse event included in the analysis was mapped to its corresponding Preferred Term in the Common Terminology Criteria for Adverse Events (CTCAE), version 5.0, to ensure standardized and reproducible classification. The following events were identified and evaluated using their CTCAE-defined terms: Liver failure, aspartate aminotransferase increased, alanine aminotransferase increased, blood bilirubin increased, hypertension, ejection fraction decreased, electrocardiogram QT corrected interval prolonged, ventricular arrhythmia, cardiac troponin I increased, stroke, transient ischemic attacks, arterial thromboembolism, laryngeal hemorrhage, upper gastrointestinal hemorrhage, lower gastrointestinal hemorrhage, hematuria, bronchopulmonary hemorrhage, epistaxis, esophageal perforation, gastric perforation, duodenal perforation, ileal perforation, small intestinal perforation, colonic perforation, hyperthyroidism, hypothyroidism, nephrotic syndrome, proteinuria, bacteremia, sepsis, pneumonitis, thrombotic thrombocytopenic purpura, hemolytic uremic syndrome, reversible posterior leukoencephalopathy syndrome, pancreatitis, retinal detachment, hair color changes, palmar-plantar erythrodysesthesia syndrome, gastric ulcer, duodenal ulcer, esophagitis, gastritis, nausea, vomiting, diarrhea, and stomach pain. In contrast, the following events were listed in the PAZ package insert but were excluded from evaluation in this study because corresponding CTCAE preferred terms could not be identified: GGT increased, angina pectoris, myocardial ischemia, cerebral hemorrhage, gastrointestinal fistula, and delayed wound healing.

Patients who discontinued hospital visits or transferred to other institutions during the follow-up period were considered censored at the date of last confirmed clinical contact and were included in analysis.

### Statistical analysis

Categorical variables were compared using Fisher’s exact test. Continuous variables were analyzed using the Mann–Whitney U test.

PFS was evaluated as the sole survival endpoint. PFS was defined as the time from the initiation of PAZ treatment to either documented disease progression or death from any cause, whichever occurred first.

In addition to the primary comparison between the AS combination group and the non-AS group, a subgroup analysis was also performed to evaluate PFS separately in patients receiving PPIs and those receiving H2RAs. Patients who received both PPIs and H2RAs during the observation period were excluded from the subgroup analysis.

Survival analysis was conducted using the Kaplan–Meier method, and differences in PFS between the AS combination group and the non-AS group were assessed using the log-rank test. A *p*-value < 0.05 was considered statistically significant.

To adjust for confounding, a Cox proportional hazards model was used, incorporating variables with known relevance to PFS in soft tissue sarcoma—PS, recurrence status, line of therapy, sex, and AS combination. PS, recurrence, and line of therapy were selected based on findings by Nassif et al. [13], while sex was included based on population-based data from Müller et al. suggesting sex-related prognostic heterogeneity [14].

All statistical analyses were performed using EZR version 1.61 [15].

## Results

A total of 99 patients who were prescribed PAZ at our hospital between November 1, 2012 and January 31, 2020, were included in this study. Among them, 77 patients were classified into the AS combination group and 22 patients into the non-AS group (Table 1).

**Table 1:** Patient background

		AS Combination Group (*n* = 77)	non-AS group (*n* = 22)	*P*
Age		54(17–74)	58(28–81)	0.393^a^
Sex	Female	59(76.6)	21(95.5)	0.064^b^
	Male	18(23.4)	1(4.5)	
Height		159(144.9–177.4)	154.6(145.5–164.0)	0.013^a^
Weight		54.3(36.9- 86.4)	53.3 (41.7- 72.2)	0.755^a^
BMI		22.1(14.8–30.0)	22.1(16.7–31.4)	0.424^a^
PS	0–1	58(75.3)	19(86.4)	0.387^b^
	2≧	19(24.7)	3(13.6)	
Recurrence	Present	58(75.3)	19(86.4)	0.387^b^
	Absent	19(24.7)	3(13.6)	
First-line therapy		13 (16.9)	5 (22.7)	0.540^b^
Histological Classification	Leiomyosarcoma	43 (55.8)	11 (50.0)	0.52^b^
	Myxofibrosarcoma	5 (6.5)	0 (0.0)	
	Undifferentiated Sarcoma	2 (2.6)	1 (4.5)	
	Liposarcoma	3 (3.9)	0 (0.0)	
	Synovial Sarcoma	2 (2.6)	0 (0.0)	
	Others	22 (28.6)	10 (45.5)	
Concomitant Medications	Lansoprazole	13 (81.2)	-	NA
	Omeprazole	2 (12.5)	-	
	Rabeprazole	1 (6.2)	-	
	Famotidine	20 (31.2)	-	
	Lafutidine	44 (68.8)	-	
dosage of pazopanib		400(200–800)	400(200–800)	0.257^a^
a history of gastrointestinal disorders		14 (18.1)	3(13.6)	0.756^b^
Usage	between breakfast and lunch	17 (22.1)	8 (36.4)	0.114^b^
	between lunch and dinner	7 ( 9.1)	5 (22.7)	
	between dinner and bedtime	19 (24.7)	5 (22.7)	
	Bedtime	27 (35.1)	4 (18.2)	
	Other	7 ( 9.1)	0 ( 0.0)	
CYP3A4 inhibitors	ketoconazole	0 (0.0)	0 (0.0)	NA
	itraconazole	0 (0.0)	0 (0.0)	
	clarithromycin	0 (0.0)	0 (0.0)	
	ritonavir	0 (0.0)	0 (0.0)	
	voriconazole	0 (0.0)	0 (0.0)	
CYP3A4 inducers	rifampin	0 (0.0)	0 (0.0)	
	carbamazepine	0 (0.0)	0 (0.0)	
	phenytoin	0 (0.0)	0 (0.0)	

Values are expressed as median (range) or n (%)

^a^Mann-Whitney U test

^b^Fisher’s exact test

Regarding PAZ administration, 82 patients (82.8%) received the standard dose of 800 mg once daily, while 17 patients (17.2%) received reduced doses (200–600 mg) based on clinical judgment or patient tolerance. In terms of dosing schedule, PAZ was most commonly administered between meals: 68 patients (68.7%) took it between breakfast and lunch, 12 patients (12.1%) between lunch and dinner, and 19 patients (19.2%) at bedtime.

Baseline characteristics between the AS combination and non-AS groups were compared. A statistically significant difference was observed only in height (*P* < 0.05), while no significant differences were found in weight, age, sex, first-line therapy, recurrence status, treatment history, or history of gastrointestinal disorders. The number of patients with a history of gastrointestinal disorders was 14 (18.1%) in the AS combination group and 3 (13.6%) in the non-AS group (*P* = 0.756).

None of the patients received concomitant medications classified as strong CYP 3A4 inhibitors or inducers during the observation period.

**Fig. 1:**
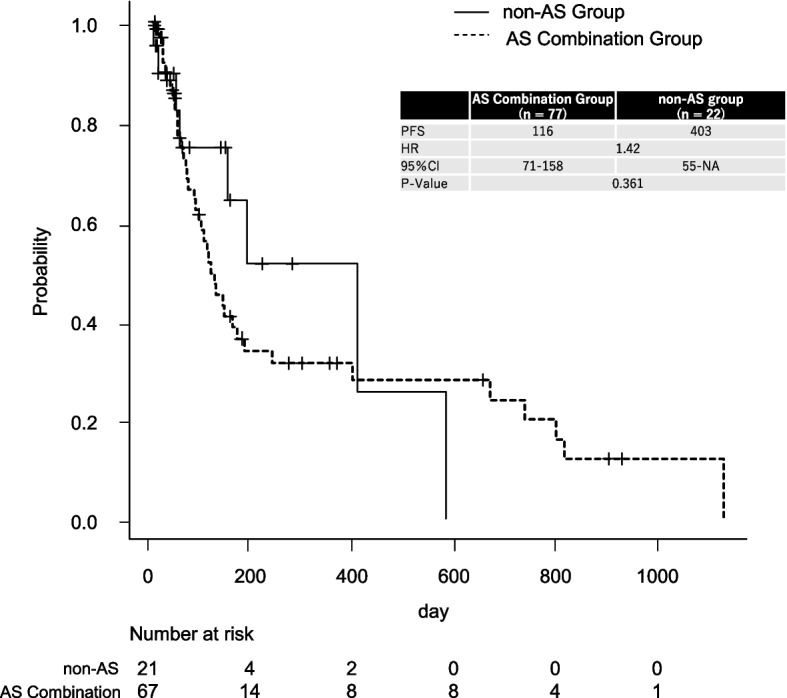
Kaplan–Meier Curves for PFS in Patients Treated With Pazopanib With or Without Acid Suppressants. The solid line represents the non-AS group (*n* = 22), and the dashed line represents the AS combination group (*n* = 77). The median PFS was 403 days in the non-AS group and 116 days in the AS combination group. The difference was not statistically significant (hazard ratio [HR] = 1.42; 95% confidence interval [CI]: 0.68–2.85; log-rank *p* = 0.361). The table inset shows the PFS and number at risk at selected time points for both groups

Kaplan–Meier curves were generated to compare PFS between the AS combination and non-AS groups (Fig. 1).

The median PFS was 116 days (95% confidence interval [CI], 71–158 days) in the AS combination group and 403 days (95% CI, 55–not available) in the non-AS group. Although the non-AS group exhibited a numerically longer median PFS, the difference was not statistically significant according to the log-rank test (*P* = 0.361).

A multivariate Cox proportional hazards regression model was used to evaluate PFS while adjusting for key prognostic factors, including AS combination, performance status, sex, first-line therapy, and recurrence status (Table 2).

**Table 2:** Multivariate cox proportional hazards analysis of PFS in patients treated with pazopanib with or without AS

	Hazard Ratio	95% Confidence Interval	*P*
AS Combination	1.20	0.54–2.63	0.66
PS	1.20	0.60–2.36	0.60
Sex	2.14	1.04–4.36	0.03*
Recurrence Status	0.89	0.49–1.62	0.71
First-line therapy	1.10	0.55–2.17	0.79

*AS* Gastric acid suppressant, *PS* Performance status

^*^P < 0.05

Among these covariates, sex was found to be a significant predictor of PFS (hazard ratio [HR], 2.20; 95% confidence interval [CI], 1.11–4.35; *P* = 0.02), indicating that male patients had a higher risk of disease progression. In contrast, concomitant use of PAZ and AS was not significantly associated with PFS (HR, 1.34; 95% CI, 0.73–2.47; *P* = 0.34), suggesting that AS combination did not independently impact treatment outcomes in this cohort.

**Fig. 2:**
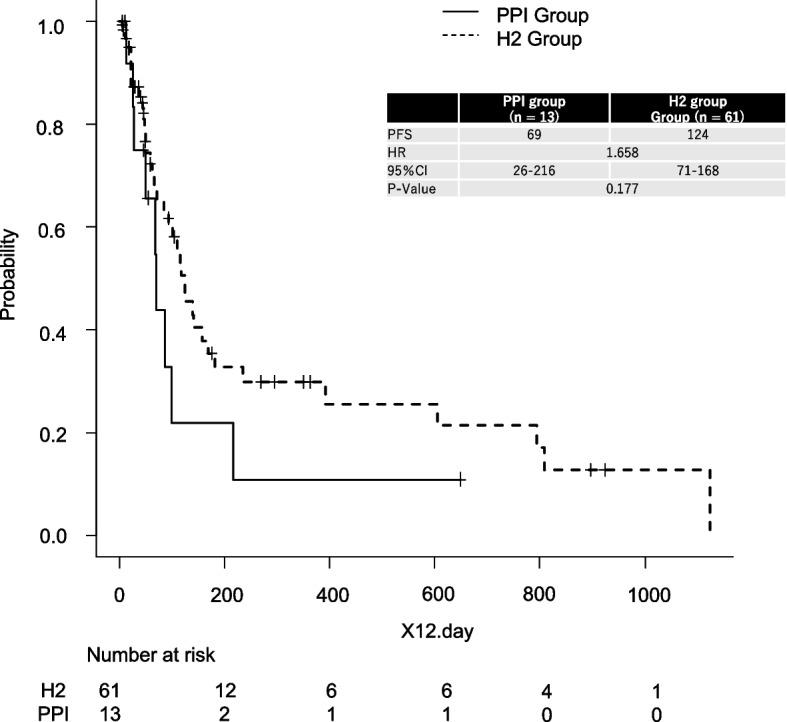
Kaplan–Meier Curves for PFS in Patients Receiving Pazopanib With Either PPIs or H2RAs. The solid line represents the PPI group (*n* = 13), and the dashed line represents the H2RA group (*n* = 61). The median PFS was 69 days in the PPI group and 124 days in the H2RA group. The difference was not statistically significant (hazard ratio [HR] = 1.658; 95% confidence interval [CI]: 0.78–3.53; log-rank *p* = 0.177). The table inset summarizes key survival metrics and number at risk at selected time points for both groups

In a subgroup analysis limited to patients receiving acid suppressants, PFS was compared between those treated with PPIs (*n* = 13) and those treated with H2RAs (*n* = 61). Patients who received both PPIs and H2RAs during the treatment period (*n* = 3) were excluded from this analysis to avoid confounding effects of overlapping acid suppression. As shown in Fig. 2, the Kaplan–Meier curves demonstrated no statistically significant difference in PFS between the PPI and H2RA groups (log-rank *P* = 0.177). Median PFS values could not be reliably estimated due to wide confidence intervals and censored data in both subgroups. However, a trend toward longer PFS in the H2RA group was observed. These findings may suggest that the impact of H2RAs on PAZ absorption is less pronounced compared to PPIs, consistent with previous pharmacokinetic studies.

During the study period, AEs were observed in 84.4% (65/77) of patients in the AS combination group and 68.2% (15/22) of patients in the non-AS group.

In terms of severity, a total of 33 grade ≥ 3 AEs were recorded in the AS combination group, compared with 9 in the non-AS group. The most frequently observed grade ≥ 3 AEs in the AS group were hypertension (*n* = 7), proteinuria (*n* = 5), and elevated ALT levels (*n* = 4). In contrast, the most common grade ≥ 3 AEs in the non-AS group were elevated ALT (*n* = 2) and proteinuria (*n* = 2). No grade 5 AEs were reported in either group.

Gastrointestinal toxicities, including gastric ulcers, duodenal ulcers, esophagitis, and gastritis, were not observed in either group. However, epigastric pain was more frequently reported in the AS combination group (18.2%, 14/77) than in the non-AS group (9.1%, 2/22). (Table 3).

**Table 3:** Adverse events

Drug insert	CTCAE v5.0	AS Combination Group (*n* = 77)						AS Non-Combination Group (*n* = 22)					
Serious Adverse Events	CTCAE v5.0 Term	All grades	Grade 1	Grade 2	Grade 3	Grade 4	Grade 5	All grades	Grade 1	Grade 2	Grade 3	Grade 4	Grade 5
Liver failure	Liver failure	0 (0%)	-	-	0 (0%)	0 (0%)	0 (0%)	0 (0%)	-	-	0 (0%)	0 (0%)	0 (0%)
AST increased	Aspartate aminotransferase increased	16(20.8%)	8(10.4%)	5(6.5%)	3 (3.9%)	0 (0%)	-	3(13.6%)	0 (0%)	2(9.1%)	0 (0%)	1 (4.5%)	-
ALT increased	Alanine aminotransferase increased	15(19.5%)	10(13.0%)	2(2.6%)	3(3.9%)	0 (0%)	-	2(9.1%)	0 (0%)	1(4.5%)	0 (0%)	1(4.5%)	-
Blood bilirubin increased	Blood bilirubin increased	8(10.4%)	5(6.5%)	2(2.6%)	1(1.3%)	0 (0%)	-	0 (0%)	0 (0%)	0 (0%)	0 (0%)	0 (0%)	-
GGT increased	-	-	-	-	-	-	-	-	-	-	-	-	-
Hypertension, hypertensive crisis	Hypertension	48(62.3%)	4(5.2%)	31(40.3%)	13(16.9%)	0 (0%)	0 (0%)	9(40.9%)	2(9.1%)	4(18.2%)	3(13.6%)	0 (0%)	0 (0%)
Left ventricular systolic dysfunction	Ejection fractiondecreased	1(1.3%)	-	1(1.3%)	0 (0%)	0 (0%)	-	0 (0%)	-	0 (0%)	0 (0%)	0 (0%)	-
QT corrected interval prolonged	ElectrocardiogramQT correctedinterval prolonged	0 (0%)	0 (0%)	0 (0%)	0 (0%)	0 (0%)	-	0 (0%)	0 (0%)	0 (0%)	0 (0%)	0 (0%)	-
Ventriculararrhythmia	Ventriculararrhythmia	1(1.3%)	1(1.3%)	0 (0%)	0 (0%)	0 (0%)	0 (0%)	0 (0%)	0 (0%)	0 (0%)	0 (0%)	0 (0%)	0 (0%)
Cardiac troponin Iincreased	Cardiac troponin Iincreased	0 (0%)	-	0 (0%)	0 (0%)	0 (0%)	0 (0%)	0 (0%)	-	0 (0%)	0 (0%)	0 (0%)	0 (0%)
Angina pectoris	-	-	-	-	-	-	-	-	-	-	-	-	-
Ischemic stroke	Stroke	0 (0%)	0 (0%)	0 (0%)	0 (0%)	0 (0%)	0 (0%)	0 (0%)	0 (0%)	0 (0%)	0 (0%)	0 (0%)	0 (0%)
Transient ischemic attacks	Transient ischemic attacks	0 (0%)	0 (0%)	0 (0%)	-	-	-	0 (0%)	0 (0%)	0 (0%)	-	-	-
Myocardial ischemia	-	-	-	-	-	-	-	-	-	-	-	-	-
Venous thrombosis, pulmonary embolism	Arterialthromboembolism	0 (0%)	-	-	0 (0%)	0 (0%)	0 (0%)	0 (0%)	-	-	0 (0%)	0 (0%)	0 (0%)
Cerebral hemorrhage	-	-	-	-	-	-	-	-	-	-	-	-	-
Hemoptysis	Laryngealhemorrhage		2(2.6%)	1(1.3%)	0 (0%)	0 (0%)	0 (0%)	1(4.5%)	1(4.5%)	0 (0%)	0 (0%)	0 (0%)	0 (0%)
Gastrointestinal bleeding	Uppergastrointestinalhemorrhage	0 (0%)	0 (0%)	0 (0%)	0 (0%)	0 (0%)	0 (0%)	1(4.5%)	0 (0%)	0 (0%)	1(4.5%)	0 (0%)	0 (0%)
	Lowergastrointestinalhemorrhage	0 (0%)	0 (0%)	0 (0%)	0 (0%)	0 (0%)	0 (0%)	0 (0%)	0 (0%)	0 (0%)	0 (0%)	0 (0%)	0 (0%)
Hematuria	Hematuria	3(3.9%)	0 (0%)	0 (0%)	3(3.9%)	0 (0%)	0 (0%)	0 (0%)	0 (0%)	0 (0%)	0 (0%)	0 (0%)	0 (0%)
Bronchopulmonaryhemorrhage	Bronchopulmonaryhemorrhage	0 (0%)	0 (0%)	0 (0%)	0 (0%)	0 (0%)	0 (0%)	0 (0%)	0 (0%)	0 (0%)	0 (0%)	0 (0%)	0 (0%)
Epistaxis	Epistaxis	10(13.0%)	10(13.0%)	0 (0%)	0 (0%)	0 (0%)	0 (0%)	0 (0%)	0 (0%)	0 (0%)	0 (0%)	0 (0%)	0 (0%)
Gastrointestinal perforation	Esophagealperforation	0 (0%)	-	0 (0%)	0 (0%)	0 (0%)	0 (0%)	0 (0%)	-	0 (0%)	0 (0%)	0 (0%)	0 (0%)
	Gastric perforation	0 (0%)	-	0 (0%)	0 (0%)	0 (0%)	0 (0%)	0 (0%)	-	0 (0%)	0 (0%)	0 (0%)	0 (0%)
	Duodenalperforation	0 (0%)	-	0 (0%)	0 (0%)	0 (0%)	0 (0%)	0 (0%)	-	0 (0%)	0 (0%)	0 (0%)	0 (0%)
	Ileal perforation	0 (0%)	-	0 (0%)	0 (0%)	0 (0%)	0 (0%)	0 (0%)	-	0 (0%)	0 (0%)	0 (0%)	0 (0%)
	Small intestinalperforation	0 (0%)	-	0 (0%)	0 (0%)	0 (0%)	0 (0%)	0 (0%)	-	0 (0%)	0 (0%)	0 (0%)	0 (0%)
	Colonic perforation	0 (0%)	-	0 (0%)	0 (0%)	0 (0%)	0 (0%)	0 (0%)	-	0 (0%)	0 (0%)	0 (0%)	0 (0%)
Gastrointestinal fistula	-	-	-	-	-	-	-	-	-	-	-	-	-
Thyroid dysfunction	Hyperthyroidism	0 (0%)	0 (0%)	0 (0%)	0 (0%)	0 (0%)	0 (0%)	0 (0%)	0 (0%)	0 (0%)	0 (0%)	0 (0%)	0 (0%)
	Hypothyroidism	23(29.9%)	1(1.3%)	22(28.6%)	0 (0%)	0 (0%)	0 (0%)	4(18.2%)	0 (0%)	4(18.2%)	0 (0%)	0 (0%)	0 (0%)
Nephrotic syndrome	Nephrotic syndrome	0 (0%)	-	-	0 (0%)	0 (0%)	0 (0%)	0 (0%)	-	-	0 (0%)	0 (0%)	0 (0%)
Proteinuria	Proteinuria	6(7.8%)	1(1.3%)	1(1.3%)	4(5.2%)	-	-	3(13.6%)	0 (0%)	1(4.5%)	2(9.1%)	-	-
Infectious disease	Bacteremia	0 (0%)	-	0 (0%)	-	-	-	0 (0%)	-	0 (0%)	-	-	-
	Sepsis	0 (0%)	-	-	0 (0%)	0 (0%)	0 (0%)	1(4.5%)	-	-	1(4.5%)	0 (0%)	0 (0%)
Delayed wound healing	-	-	-	-	-	-	-	-	-	-	-	-	-
Interstitial pneumonia	Pneumonitis	2(2.6%)	0 (0%)	0 (0%)	2(2.6%)	0 (0%)	0 (0%)	0 (0%)	0 (0%)	0 (0%)	0 (0%)	0 (0%)	0 (0%)
Thrombophilia	Thromboticthrombocytopenicpurpura	1(1.3%)	-	-	1(1.3%)	0 (0%)	0 (0%)	-	-	-	0 (0%)	0 (0%)	0 (0%)
	Hemolytic uremicsyndrome	0 (0%)	-	-	0 (0%)	0 (0%)	0 (0%)	0 (0%)	-	-	0 (0%)	0 (0%)	0 (0%)
Reversible posteriorleukoencephalopathy syndrome	Reversible posteriorleukoencephalopathy syndrome	0 (0%)	-	0 (0%)	0 (0%)	0 (0%)	0 (0%)	0 (0%)	-	0 (0%)	0 (0%)	0 (0%)	0 (0%)
Pancreatitis	Pancreatitis	2(2.6%)	-	1(1.3%)	1(1.3%)	0 (0%)	0 (0%)	0 (0%)	-	0 (0%)	0 (0%)	0 (0%)	0 (0%)
Retinal detachment	Retinal detachment	0 (0%)	-	-	0 (0%)	0 (0%)	0 (0%)	0 (0%)	-	-	0 (0%)	0 (0%)	0 (0%)
Hair color changes	Hair color changes	11(14.3%)	11(14.3%)	-	-	-	-	0 (0%)	0 (0%)	-	-	-	-
Palmar-plantarerythrodysesthesiasyndrome	Palmar-plantarerythrodysesthesiasyndrome	16(20.8%)	14(18.2%)	2(2.6%)	0 (0%)	-	-	5(22.7%)	5(22.7%)	0 (0%)	0 (0%)	-	-
Gastric ulcer	Gastric ulcer	0 (0%)	0 (0%)	0 (0%)	0 (0%)	0 (0%)	0 (0%)	0 (0%)	0 (0%)	0 (0%)	0 (0%)	0 (0%)	0 (0%)
Duodenal ulcer	Duodenal ulcer	0 (0%)	0 (0%)	0 (0%)	0 (0%)	0 (0%)	0 (0%)	0 (0%)	0 (0%)	0 (0%)	0 (0%)	0 (0%)	0 (0%)
Reflux esophagitis	Esophagitis	0 (0%)	0 (0%)	0 (0%)	0 (0%)	0 (0%)	0 (0%)	0 (0%)	0 (0%)	0 (0%)	0 (0%)	0 (0%)	0 (0%)
Acute gastritis, chronic gastritis	Gastritis	0 (0%)	0 (0%)	0 (0%)	0 (0%)	0 (0%)	0 (0%)	0 (0%)	0 (0%)	0 (0%)	0 (0%)	0 (0%)	0 (0%)
Nausea	Nausea	21(27.3%)	11 (14.3%)	9 (11.7%)	1 (1.3%)	-	-	3(13.6%)	2 (9.1%)	1(4.5%)	0 (0%)	-	-
Vomiting	Vomiting	11(14.3%)	5(6.5%)	5(6.5%)	1 (1.3%)	0 (0%)	0 (0%)	4 (18.2%)	1(4.5%)	3(13.6%)	0 (0%)	0 (0%)	0 (0%)
Diarrhea	Diarrhea	19(24.7%)	15(19.5%)	4(5.2%)	0 (0%)	0 (0%)	0 (0%)	2 (9.1%)	1(4.5%)	1(4.5%)			
Stomach pain	Stomach pain	14 (18.2%)	12 (15.6%)	2 (2.6%)	0 (0%)	-	-	2 (9.1%)	2 (9.1%)	0 (0%)	0 (0%)	-	-

n (%)

Items without classification are indicated as “-”

## Discussion

This study found no statistically significant difference in PFS between Japanese sarcoma patients receiving PAZ with AS and those without, although a numerically longer median PFS was observed in the non-AS group (403 vs. 116 days). This finding should be interpreted with caution. The non-AS group included only 22 patients, and a considerable proportion of them were censored due to relatively fewer events (i.e., progression or death) during the observation period, which may have artificially prolonged the estimated median PFS. One potential explanation for the baseline differences is the imbalance in patient characteristics; the AS combination group included a higher proportion of male patients, which may reflect underlying differences in disease types and comorbidities such as gastroesophageal reflux disease that necessitate AS use [16, 17]. While the multivariate analysis adjusting for these confounding factors, including sex, found no significant effect of AS combination on PFS, the substantial numerical difference observed warrants serious clinical consideration. Especially in a rare disease context like sarcoma, such a large difference suggests that even modest interactions may be highly relevant in real-world settings.

In the subgroup analysis, no statistically significant difference in PFS was observed between the PPI group and the H2RA group (*P* = 0.177). However, it is important to recognize that PPIs have a greater potential to raise gastric pH and reduce PAZ absorption compared to H2RAs, as shown in previous pharmacokinetic studies. The lack of significant differences in our study may reflect the limited sample size and the inherently short prognosis of advanced soft tissue sarcoma, which may mask subtle pharmacokinetic effects over longer treatment durations.

In terms of safety, an unexpected finding was the trend towards a higher frequency of dose-dependent adverse events like hypertension and hepatotoxicity in the AS group. While this may suggest altered exposure profiles potentially driven by pH-mediated absorption issues, this observation is paradoxical. Given that AS agents are hypothesized to decrease pazopanib absorption, a corresponding decrease in dose-dependent toxicities would be expected, not an increase. A more plausible explanation is that this finding may reflect potential underlying comorbidities or gastrointestinal conditions rather than a direct effect of AS on PAZ toxicity. Although epigastric pain was more common in the AS combination group, it did not lead to treatment discontinuation. Based on these findings, the use of AS for PAZ-induced gastrointestinal toxicities may help mitigate the severity of symptoms, prevent severe AEs such as gastrointestinal bleeding, and thereby reduce treatment discontinuation rates.

Several other factors could explain why a clinically significant effect of AS was not statistically proven in this cohort. The effect of AS on PAZ exposure may be less pronounced in real-world clinical settings than in controlled pharmacokinetic studies due to factors like diet, counseling on administration practices, and patient adherence. This discrepancy may also be attributable to inter-individual variability in drug metabolism or the fact that the therapeutic window of PAZ allows for some variability in systemic exposure without significantly affecting efficacy. Furthermore, while other drugs like strong CYP3A4 inhibitors and inducers are known to affect PAZ pharmacokinetics, no patients in our cohort received these agents, so their impact could not be assessed.

When considering our findings, it is also important to note potential differences between populations. While key pharmacokinetic parameters for pazopanib appear generally comparable between Japanese and Western populations [10, 18, 19], differences in safety profiles have been observed. Asian patients have shown a higher incidence of AEs such as hypertension, elevated liver enzymes, and hematologic toxicity, while Western patients more frequently experience gastrointestinal symptoms [18]. These discrepancies may reflect genetic factors (e.g., HIF1A polymorphisms [20]), as well as cultural or dietary practices, such as adherence to fasting conditions and prevalence of high-fat meals. Therefore, careful consideration of patient-specific factors is essential to optimize treatment outcomes in real-world clinical settings.

This study has several limitations. The retrospective nature of the study limits the ability to establish causal relationships and is susceptible to residual confounding, although we adjusted for key prognostic variables. Furthermore, the relatively small sample size, particularly in the non-AS group, may have reduced the statistical power to detect a true difference in PFS. A more significant source of potential bias relates to unmeasured factors regarding drug administration, which would likely bias our findings toward the null. We could not assess actual patient adherence to either pazopanib or the AS agents. Non-adherence to oral oncolytics is common and would lead to sub-therapeutic drug exposure, likely attenuating any observable negative effect of concomitant AS use. Similarly, the precise timing of AS administration relative to pazopanib could not be confirmed. Given that the interaction is pH- and time-dependent, this uncontrolled variable introduces a significant potential confounder. Collectively, these limitations—non-adherence, variability in administration timing, and potential misclassification from unrecorded OTC use—mean that the true detrimental effect of concomitant AS use may be greater than what we observed. Finally, the absence of therapeutic drug monitoring means we have no pharmacokinetic data to directly assess the impact of AS co-administration on pazopanib plasma concentrations in our cohort.

From a clinical perspective, while our findings suggest that AS combination may not drastically compromise PAZ efficacy, interpretation requires caution. The use of acid suppressants—particularly PPIs—should be carefully considered. While the use of AS may be justifiable in patients experiencing significant gastrointestinal symptoms or receiving antiplatelet/anticoagulant therapy, indiscriminate or prolonged use without clear indication should be avoided. Clinical decision-making should remain individualized.

## Conclusions

In our cohort of Japanese patients with sarcoma, the concomitant use of a gastric AS with PAZ was not associated with a statistically significant difference in PFS. This finding, however, must be interpreted with extreme caution. The substantial numerical difference in median PFS—116 days in the AS group versus 403 days in the non-AS group—suggests a potentially clinically meaningful negative effect that our study was likely underpowered to detect.

Therefore, our results do not prove the absence of a detrimental interaction. This lack of statistical significance may be attributable to key study limitations, including the retrospective design, a small sample size (particularly in the non-AS group), and a lack of data on patient adherence and administration timing.

These findings underscore the importance of individualized patient care. While the co-administration of AS may be reasonable under careful monitoring for patients with a strong clinical indication, such as managing gastrointestinal symptoms, indiscriminate or prolonged use without a clear purpose should be avoided given the potential to compromise PAZ efficacy.

To definitively resolve this issue, future prospective, multicenter studies incorporating pharmacokinetic assessments are essential to validate these findings and to guide optimized treatment strategies for patients with sarcoma requiring AS therapy.

## Data Availability

All data generated or analyzed during this study are included in this published article.

## Abbreviations

PAZ: Pazopanib hydrochloride
PPI: Proton pump inhibitor
H2RA: Histamine H2 receptor antagonist
AEs: Adverse Events
AS: Gastric acid suppressant
PFS: Progression-free survival
OS: Overall survival
BMI: Body mass index
CYP: Cytochrome P450
PS: Performance status
OTC: Over-the-counter
AST: Aspartate Aminotransferase
ALT: Alanine Aminotransferase
GGT: Gamma-Glutamyl Transferase
CTCAE: Common Terminology Criteria for Adverse Events
HR: Hazard ratio
CI: Confidence interval
